# Cervical pre-cancer screening by visual inspection of the cervix after application of acetic acid in rural Burkina Faso: evaluation of women’s knowledge, screening practice habits, acceptability and prevalence of risk factors and lesions in Boussé health district

**DOI:** 10.11604/pamj.2023.45.135.36933

**Published:** 2023-07-19

**Authors:** Souleymane Tassembedo, Christian Henrik Winter, Isidore Tiandiogo Traore, Adama Ouattara, Mamadou Sawadogo, Nicolas Meda

**Affiliations:** 1 Centre MURAZ Research Institute, Infectious Diseases Research Programme, Bobo-Dioulasso, Burkina Faso,; 2 Robert Koch Institute, Berlin, Germany,; 3 Nazi Boni University, Department of Public Health, Bobo-Dioulasso, Burkina Faso,; 4 Centre for International Research in Health, University of Ouagadougou, Burkina Faso,; 5 Yalgado Ouedraogo Research and Training Hospital, Department of Gynecology and Obstetrics, Ouagadougou, Burkina Faso,; 6 University of Ouagadougou, Department of Public Health, Ouagadougou, Burkina Faso

**Keywords:** Cervical pre cancer, screening, rural women, Burkina Faso

## Abstract

**Introduction:**

cervical cancer is a major public health problem among women in sub-Saharan Africa. The disease can be controlled through early diagnosis through simple cost-effective methods such as visual inspection of the cervix after application of acetic acid or lugol´s iodine. However, screening for cervical cancer is still underused particularly in rural areas of Burkina Faso. The objective was to estimate the prevalence of cervical pre-cancer cancer in rural health district of Boussé, Burkina Faso.

**Methods:**

we conducted a cross-sectional study in the health district of Boussé in Northern-Central Burkina Faso from July to August 2014. Women aged 23-50 years were interviewed about their knowledge of cervical cancer and their screening practice and subsequently screened for cervical cancer by VIA.

**Results:**

a total of 418 participants were included with a median age of 34 years IQR (30-40 years). Two^2^ hundred participants (48%) had never heard about cervical cancer. About 134 participants (32%) knew at least one risk factor of cervical cancer. Only 37 women (9%) reported ever being screened for cervical cancer. Twenty-two percent reported concurrent sexual partnerships. The majority of the women (92%) are willing to pay to get screened for cervical pre-cancer by VIA. Overall, 21 participants (5%) were diagnosed with a cervical lesion by VIA and all of them accepted treatment with Loop electro surgical procedure.

**Conclusion:**

screening by VIA is feasible in rural Burkina Faso, but there is a poor knowledge on cervical cancer amongst the women. There is a need to set up a comprehensive, systematic, affordable and efficient cervical cancer program including an information campaign and making screening accessible in rural remote areas.

## Introduction

Cervical cancer is a major global public health issue. It is the second most common cancer in women worldwide with 500,000 new cases and 250,000 deaths annually, with about 80% of the new cases occurring in developing countries [1-3]. In 2009, the estimated incidence of cervical cancer was 1,230 per year resulting in 838 deaths in Burkina Faso [4]. A retrospective study conducted in 2008 in the major cytology and pathology laboratories of the country, showed that cervical cancer represented the second most common cancer in women [5]. Cervical cancer is curable if diagnosed and treated early thanks to its slow development that could last up to ten years. In most developed countries, comprehensive strategies for cervical cancer management are available including strong educational programs, immunization against high risk human papillomavirus (HPVs), and screening of women and treatment of cervical cancer cases [4]. As a result, there has been a dramatic reduction of the cervical cancer incidence in middle income countries in recent years [2]. Conversely, in developing countries there is a persistent lack of efficient programs, suitable facilities and well-trained personnel to deal with the increasing toll of cervical cancer [1]. The situation is similar in Burkina Faso where about one third of the patients were diagnosed in advanced stages of the disease [6] leading to poor treatment outcomes. Thus, cervical cancer screening plays a major role in the strategies to detect cervical cancer early and control it. A range of tools with different degrees of accuracy including human papillomavirus (HPV) DNA testing, Pap smear, and visual inspections exist. But their implementation depends on the availability of required materials as well as trained human resources in each country. Visual inspections, an alternative screening method is widely implemented in most low- and middle-income countries (LMICs) including sub-Saharan Africa. Studies in Africa, Asia, and Latin America supported Visual Inspection of the cervix after application of Acetic acid (VIA) for cervical pre-cancer screening in low and middle-income countries (LMIC) [7-10]. WHO suggested alternative screening tools such as Visual inspection of the cervix after application of acetic acid (VIA) to be used for screening purposes in low-income settings [4]. Since 2013, WHO has recommended, human papillomavirus testing as the preferred primary screening tool with or without VIA [11]. In Burkina Faso, the implementation of the cervical cancer prevention (CECAP) program between 2010 and 2014 [12] boosted alternative screening techniques such as VIA, this improved the coverage of cervical prevention services in the country. This large study including 14 health facilities found a prevalence of VIA positive lesions of 8.9% [12]. But its sustainability by and expansion within the care services remained a challenge [12,13]. Cervical cancer screening by VIA/VILI is mostly delivered by public health facilities from the tertiary (teaching hospitals) and secondary (regional hospitals) level of the health care system. In addition, some district hospitals mainly from urban settings also provide such screenings. Moreover, mass opportunistic and sporadic screening campaigns (e.g. during Women´s Day) are usually conducted in urban areas. The rural communities are underserved and little is known about the prevalence of cervical pre-cancer among women in these settings. Therefore, an assessment of cervical cancer screening in rural Burkina Faso was needed. The purpose of this study was to estimate the prevalence of cervical pre-cancer cancer in rural health district of Boussé, Burkina Faso in order to improve it if necessary.

## Methods

**Study design**: we conducted a cross-sectional study from June to July 2014.

**Setting**: the study was conducted in the rural Health District (HD) of Boussé, which is located about 50km North of Ouagadougou in the North Central region. Five screening points were available including four in primary health facilities (Laye, Niou, Toeghin and Sourgoubila) and at the District hospital (Boussé).

**Participants**: women between 23 and 50 years inhabiting the catchment of the Boussé health district were eligible. Pregnant women, women with active vaginal bleeding, women with abnormal growth on the cervix, women who never had sexual intercourse, women who had undergone a hysterectomy or prior treatment for any kind of cervical neoplasm were not eligible for this study. Women attending the 5 health facilities involved in the study were offered voluntary participation in cervical pre-cancer screening using VIA tests during their visits.

**Variables**: the main outcome variable is the VIA test result who performed the testing (Positive/Negative). The sociodemographic variables include: age, education, occupation and main source of income. The exposure variables are: age at sexual debut, ever heard about cervical cancer, knowledge of preventive measures and ever been screened for cervical cancer.

### Data sources/measurement

**Study procedures**: gynecologists assisted by experienced and well-trained midwives were responsible for performing the VIA tests after a refresher training. An unlubricated bivalve speculum was inserted into the vagina by the midwife who examined the cervix using a halogen focus lamp and identified the squamocolumnar junction (SCJ). After cleaning away any excess mucus using a cotton swab, a 5% acetic acid solution was applied to the cervix for the VIA. Findings were visible one minute after application. A positive VIA was characterized by a well-defined, dense aceto-white area with regular or irregular margins, close to or abutting the SCJ in the transformation zone or when the whole cervix or a cervical growth turned acetowhite. Following VIA, visual inspection with Lugol´s iodine (VILI) was also performed. A positive VILI test was characterized by a dense, thick, bright, mustard-yellow or saffron-yellow iodine non-uptake area seen in the transformation zone, close to or abutting the squamous columnar junction (SCJ) or when the entire cervix or a cervical growth turns densely yellow. Results of VIA and VILI were both classified as negative, positive or indicative only of invasive cervical cancer (ICC) according to the training manual of the International Agency for Research on Cancer (IARC) [14]. All women tested VIA positive from all the different peripheral sites were referred to the central site in the health district of Boussé to be retested by a senior staff. They underwent treatment by LEEP [15] done by a qualified and experienced gynecologist within 7 days if confirmed positive. After the procedure, the women stayed at the hospital under surveillance until the evening. They were explained to immediately contact the study team should any problem occur after their admission from the hospital.

**Data collection**: medical students, who were trained as data collectors, interviewed the participants at the five sites by using a standardized questionnaire. This questionnaire collected information on sociodemographic characteristics, some risk factors of cervical cancer (age at sexual debuts, number of sexual partners…), Cervical cancer screening history and the outcome of the VIA screening. Data was entered into “Epi Info 3.5.2” using a double data entry system.

**Study size**: the minimum sample size is obtained with the below formula:


$$n=\left[DEFF*Np\left(1-p\right)\right]/\left[(d2/Z21-\alpha /2*\left(N-1\right)+p*\left(1-p\right)\right]$$

Where p (anticipated proportion of VIA positive result) =8.9% in Burkina Faso according to a large study [12], DEFF (Design effect) =2, d (confidence limit) = 5, α (the desire precision) =5%, Z_1- α/2_=1.96 and Np the overall Boussé population which is estimated at more than 500000 women. Applying this formula gives a minimum sample size of 250 women. Considering a 10% non-response among the eligible women, it arrived at a minimum study sample size of 275 women to enroll.

**Statistical analysis**: we conducted a descriptive data analysis using “Epi Info 3.5.2”. Quantitative variables have been described as median, [IQR] and qualitative variables according to their frequencies and percentages.

**Ethical considerations**: ethical approval of the study protocol was obtained from the Ministry of Health and the Ministry of Scientific Research and innovation´ Health Research Ethics Committee (2014-4-023). Written informed consent was obtained from each participant prior to her inclusion in the study. Women screened positive gave their verbal agreement to undergo the treatment by LEEP (15) as recommended by the WHO [11].

## Results

**Characteristics of the study population**: during the study period, 435 women were assessed for study participation, 7 declined participation, 5 were above 5 years and 5 were menstruating and 5 had lesions suggestive of infections and were not included overall, 418 met the inclusion criteria and were screened for cervical pre-cancer. The median age of the study participants was 34 years (IQR 30-40 years). The median age for the first sexual intercourse was 19 years (IQR 17-19 years). Housewives accounted for 44.7% of the participants and 59.6% of the participants have not received any formal education (Table 1).

**Table 1:** socio-demographic characteristics of the study participants (N=418)

Characteristics	Median [IQR]	n (%)
Age, years	34[30-40]	
Village of origin		
Boussé		145(34.7)
Laye		108(25.8)
Sourgoubila		91(21.8)
Niou		27(6.5)
Toeghin		47(11.2)
Occupation		
Housewives		100(23.9)
Vendor		59(14.1)
Government/private employees		166(39.7)
Farmers		72(17.2)
Other		21(5)
Education level		
None		249(59.6)
Primary school		60 (14.3)
Secondary school		101(24.2)
University		8(1.9)
Main income source		
From the partner		180(43.1)
Personal		199(47.6)
From siblings		29(6.9)
Other		10(2.4)

n (%): number (percentage)

**Human resources for VIA and knowledge of the participants regarding cervical cancer**: in the health district of Boussé, two midwives were adequately trained to perform VIA tests. About half of the participants (200/412) had never heard about cervical cancer. Ten women were aware of the causative agent of cervical cancer. Eighteen women (4%) could show the real location of the cervix on a diagram. Thirty-two percent of the participants knew at least one of the risk factors of cervical cancer and 35% knew at least one preventive measure against cervical cancer (Table 2).

**Table 2:** study participants knowledge regarding cervical cancer and practice of cervical cancer screening (N=418)

Knowledge	n (%)
Never heard about cervical cancer *	200(49.0)
Localized correctly the cervix on a diagram+	18(4.0)
Knew at least one risk factor of CC	134(32.0)
Knew at least one preventive measure	145 (35.0)
Knew that CC is an STI	97(23.2)
Practice of screening	
Ever been screen for CC	37(8.8)
Type of screening tool	
VIA	35(94.6)
Pap smear	2(5.4)

n (%) number (percentage); *=06 missing values; += 01 missing value

**Prevalence of cervical cancer risk factors in the study population**: the prevalence of some of the known risk factors of cervical cancer in our sample was 22% and 7% for multiple concurrent sexual partnership and high parity respectively (Table 3).

**Table 3:** cervical cancer risk factors among study participants (N=418)

Characteristics	n (%)
Age at sexual debut <15 Years*	9(2.24)
**Prolonged oral contraception** (more than 5 years)	4(1)
**Concurrent sexual partnerships**	93(22.2)
**High parity** (delivered more than 3 times)	29(6.9)
**Ever smoked**	8(1.9)

* =17 missing values

**Acceptability of cervical cancer screening by VIA and willingness to pay**: overall 98.8% of the participants (418/423) accepted to be screened for cervical pre-cancer by VIA/VILI. Five women refused to be screened after being explained all the study procedures for inconvenience with being examined nude or with a speculum About 92% of the participants (380/418) would be willing to pay for cervical pre-cancer screening: 36% (144/380) if the cost is less than 500 FCFA (1 USD), 32% (132/380) if the cost is between 500 and 1000 FCFA (1-2 USD) and 28% (99/380) even if the is more than 1000 FCFA (2 USD).

**Screening experiences and prevalence of cervical lesions by VIA**: about 9% (37/418) of the participants had undergone any cervical cancer screening by VIA/VILI or Pap smear at least once in the past. The per-speculum examination found that 17% of the participants (28/418) had an abnormal looking cervix. In total, 21 women 5%, (95% Confidence interval 2.7%-7.8%) were found with cervical pre-cancer lesions with VIA/VILI. Among them, 11 had an abnormal looking cervix and 10 had a normal looking cervix at the per speculum examination. The median age of the women with a positive VIA/VILI is 36 years IQR (32-40). All the women diagnosed with cervical lesions accepted the treatment by LEEP as proposed by the study team.

## Discussion

This cross-sectional study found that cervical pre-cancer prevalence is this rural health district was 5%. It also showed a poor availability in qualified personnel to perform screening as well as a poor knowledge of the women regarding cervical pre-cancer screening. Our estimated prevalence of VIA positive test (5%) is lower than that of a larger study in Burkina Faso (8.9%) [12]. This study is mainly conducted is urban settings where the prevalence of risk factors such as smoking or early sexual debut may be higher. The health district of Boussé has about 500,000 women of reproductive age [16], but only two midwives both working in the district capital of Boussé were trained to screen for cervical pre-cancer using VIA. The lack of qualified human resources for cervical cancer screening in low-income countries was pointed-out [17] and this hinders the provision and sustainability of regular screening by health facilities. In addition, the distance and costs to travel to Boussé could be challenging for many women. It prompts the emergency to train additional workforce for screening including professional and non-professional health care workers in a task shifting and decentralization perspectives. This will also require balancing this staff between urban and rural health facilities. The poor knowledge about cervical cancer with about 50% of the women having never heard about it was worrying. Studies conducted in Ouagadougou both population based and hospital based found that 64.2% [18] and 90% [19] of the women have heard about this disease respectively. This highlights the urban-rural divide in the access to information about cervical cancer. More importantly, only 32% knew about the transmission of the causative agent and preventive measures. The lack of accessible information and sensitization regarding this problem especially in remote rural areas could explain the situation.

Part of the problem could be the low literacy rate in the country especially among girls and women in rural areas [17]. Additional efforts need to be invested by health authorities to inform the women on cervical cancer and the availability of cervical cancer screening. Less than 10% of women had already undergone a cervical cancer screening. That is probably not only the result of poor information regarding cervical cancer (as noted above) but also the lack of adequate infrastructure and sufficiently skilled staff to perform cervical pre-cancer screening even with the alternative simple methods such as visual inspection. Our study like other screening campaigns contributed to fulfill the recommendation of the Alliance for Cervical Cancer Prevention (ACCP), which is that “every woman has the right to cervical screening at least once in her lifetime”. Although temporary, such campaigns should be sustained by the associations promoting them until a national cervical cancer screening program is implemented. With the implementation of high performance GeneXpert DNA screening assay with a sensibility of 98.3% for CIN2 lesion [20] in some health districts supported by Non-Governmental Organizations, the picture may have evolved positively. In addition this Point Of Care machinery this device has the advantage of being easy to use for the diagnosis of other diseases (HIV, Tuberculosis, some sexually transmitted diseases) provided the suitable cartridges are available. But the operational challenges related to the limited availability of the machines and trained laboratory technologists, poor power availability in remote setting, sample conservation may not allow a thorough implementation of this technique countrywide. This stress that VIA still has an important role to play in the cervical screening landscape. Concurrent sexual partnership has been reported by almost one in four women. This behavior has been linked to the high burden of some of the sexually transmitted diseases including infection by the human papillomavirus infection, the main causative agent of cervical cancer. This underlines the need for an integrated sensitization approach against all sexually transmitted diseases. High parity is also a concern in our population. This could be due to the low coverage and uptake of family planning [16] especially in remote rural areas where it is often still a taboo. Family planning should be promoted and made available for women in rural areas.

Our study included the provision of basic information about the disease at the end of the interview. This included the right information regarding its preventability and treatment options if diagnosed early and most of the women were willing to pay out of their own pockets for the testing. Alternative screening methods are cost effective and could be sustained in low-income settings [21] considering that the screening is recommended every 3-5 years. Cervical pre-cancer screening by visual inspection with acetic acid was acceptable in rural Burkina Faso with only 1.6% refusal rate (7/435). This high rate of acceptance combined with the reported high rate of willingness to pay for screening of 92% is reassuring and may suggest that the poor uptake of the screen is mostly linked to the provider side. It pledges for training more nurses to VIA as well as equipping adequately their health facilities of origin with the required materials and conducting more awareness campaigns to achieve more significant uptake. And once all these requirements are met, following thoroughly the steps described by Nene to enhance community participatory in this study could be of importance [22]. But this theoretical willingness to pay may less in real world as Ouedraogo *et al*. noticed a drop in same day cryotherapy demand when charged for 2 USD [12]. About half of the women with positive VIA had a normal looking cervix at the per-speculum examination. A study from Nepal found similar results [23] although with Pap smear as gold standard. This reinforced the importance for women to have systematic cervical cancer screening should it be by Pap smear or alternative screening methods particularly for those who are not using health services on a routine basis. The median age of the women with cervical abnormalities at the VIA/VILI tests was 36 years. This finding is consistent with those of several other studies, which find that cervical cancer is most frequent in women between 40 and 50 years given the latency of 5 to 10 years prior to the development of cervical cancer. Our study unlike one conducted in Uganda [24] showed that the low literacy of the women tested positive, did not impact negatively on their acceptance of the treatment. This might be due to the good communication process the team engaged with participants and the subsequent trust established. Also, the fact that women had the promise that they could go back home the same day could have contributed to this success.

**Limitations**: our study missed the opportunity to perform histological analysis on the samples we collected from our participants with cervical abnormalities at the VIA/VILI tests. VIA has been criticized for its low specificity by several studies [7,25]. The issues arising from this practice could be overestimating the prevalence of cervical pre-cancer and over treating women. However, this should not prevent women from low-income settings to benefit from alternative screening. Indeed, late diagnosis is associated with low survival rates and high management costs. We therefore support the use of these tests until more suitable, affordable and efficient technologies emerge. Questions exploring sexuality are taboo in our context and some women could have given social desirability bias. But we ensured confidentially during the interviews to encourage sincerity in the reports. Rural communities sometimes underuse the health facilities but we tackled it through mass communication campaign before and during the screening implementation. In total, we think our study findings are generalizable to rural communities in Burkina Faso.

## Conclusion

Five percent of the participants had pre-cancerous lesions that could have developed into life-threatening cervical cancer. Their knowledge regarding cervical cancer and its screening is low in the rural health district of Boussé. Moreover, there are few trained health workers for VIA/VILI resulting in few women having undergone a cervical cancer screening. Although their carriage of cervical cancer common risk factors is relatively high. Having a nationwide screening program including sensitization on its risk factors, training health care providers in screening and equipping rural health facilities with screening and cases management capacities in place could save thousands of women´s lives.

### What is known about this topic

- *Cervical re-cancer screening by VIA is feasible in teaching, regional, and urban districts hospitals;*
- *Women´ knowledge of cervical cancer and the prevalence of cervical pre-cancer in urban settings of Burkina Faso*.

### What this study adds

- *Women in rural settings of Burkina Faso face a low coverage of cervical cancer screening;*
- *Cervical pre-cancer screening by VIA at a primary health care facility in a rural setting in Burkina Faso is feasible;*
- *Women in these underserved settings are willing to pay to get screened for cervical pre-cancer*.
